# AKT1 and PIK3CA activating mutations in Moroccan bladder cancer patients´ biopsies and matched urine

**DOI:** 10.11604/pamj.2022.41.59.31383

**Published:** 2022-01-20

**Authors:** Hajar El Ahanidi, Meryem El Azzouzi, Housna Arrouchi, Chaimae Hafidi Alaoui, Mohammed Tetou, Mounia Bensaid, Mohamed Oukabli, Ahmed Ameur, Abderrahmane Al Bouzidi, Mohammed El Mzibri, Mohammed Attaleb

**Affiliations:** 1 Biology and Medical Research Unit, The National Center for Energy and Nuclear Science and Technology, Rabat, Morocco,; 2 Rabat Medical and Pharmacy School, Mohammed V University in Rabat, Rabat, Morocco,; 3 Military Hospital Mohammed V, Rabat, Morocco

**Keywords:** Bladder cancer, PIK3CA, AKT1, activating mutations, Moroccan patients

## Abstract

**Introduction:**

in cancer cells, activating mutations in PIK3CA and AKT1 genes, major players of PI3K-AKT-mTOR signalling pathway, are widely reported in many cancers and present attractive targets for the identification of new therapeutics and better cancer management. The present study was planned to evaluate the mutational status of PIK3CA and AKT1 genes in bladder cancer patients and to assess the association between these mutations and patients´ clinico-pathological features.

**Methods:**

in this prospective study, bladder cancer biopsies and matched urine sediments samples were collected form 70 patients. Mutations were assessed by deoxyribonucleic acid (DNA) sequencing and correlation with clinico-pathological data was performed using SPSS software.

**Results:**

AKT1 alterations were poorly detected. Only one patient with pT1 stage and high-grade tumour carried the E17K mutation. In PIK3CA exon 9, 2 point mutations, E545K and Q546E, and a SNP (E547E) were reported, whereas in exon 20, 2 point mutations (L989V and H1047R) and 2 SNPs (I1022I and T1025T) were detected. PIK3CA mutations were mainly observed in early stages and high-grade tumours. Statistical analysis showed no significant association between the studied AKT1 and PIK3CA mutations and patients´ clinico-pathological parameters (p > 0.05). Detection of these mutations in voided urine samples showed a high specificity (100%) for both genes and a moderate sensitivity: 100% for AKT1 and 66.7% for PIK3CA genes.

**Conclusion:**

this study shows clearly that mutations in AKT1 and PIK3CA are rare events and could not be considered as valuable biomarkers for bladder cancer management.

## Introduction

Bladder cancer (BC) is the ninth most commonly diagnosed cancer in the world, accounting for approximately 420,000 new cases each year [1]. In Morocco, bladder cancer is a very significant public health problem, in terms of prevalence, mortality, impact on quality of life and economic cost [2]. According to the regional cancer registers of Casablanca and Rabat, the incidence of BC reached 5.8 and 11.3 per 100,000 persons, respectively [3,4]. Of particular interest, BC is most frequently diagnosed in males and the most frequent histological type is by far transitional cell carcinoma, usually diagnosed at stages I and II [5]. The most common presentation of bladder cancer is visible, or gross, hematuria, but patients can also present with isolated microscopic hematuria. The risk of bladder cancer is approximately 4% in patients with microscopic hematuria and 16.5% in those with gross hematuria [6]. In Morocco, the treatment of BC is based on the clinic-pathologic features [7] and the current standard method for urothelial BC detection in both the incident and surveillance settings are cystoscopy and conventional urine cytology [8]. Cystoscopy is an invasive approach that provides essential prognostic information, but shows insufficient power to precisely predict the patient outcome. Cytology is a largely used non-invasive method that presents some limits, likely related to its low sensitivity for low grade tumors and the user-dependent cytological interpretation [9]. These disadvantages are underlining the urgent need to implement more reliable and objective tools to diagnose and monitor BC patients. Currently, the development of molecular biology tools has given access to explore molecular alterations in bladder cancer and identifying potential diagnostic, prognostic and therapeutic targets; and numerous promising biomarkers have been identified, including genomic alterations and transcriptional subtypes [10]. In this field, frequent activating mutations have been identified, and multiple studies have focused on hotspot mutations markers for disease surveillance using urine sediments as promising diagnostic tool for non-invasive BC diagnosis [11, 12].

In cancer cells, PI3K-AKT-mTOR signalling pathway plays a major role in growth, proliferation and cellular survival. This pathway is either activated by extracellular signals (receptors with tyrosine kinase activity) or by intracellular ones through the transformation or the over expression of proteins involved in the signal transduction [13, 14]. In BC, 50-70% of tumours contain mutations that are predicted to activate this pathway, including activating mutations in PIK3CA, a member of PI3K family, and in AKT1 genes [15]. PI3Ks (Phosphoinositide 3-kinases) are a class of enzymes that phosphorylate a series of membrane phospholipids playing a crucial role in tumour occurrence as part of the PI3K-AKT-mTOR signalling pathway [16]. In many cancers, a great interest has been focused on the identification of new inhibitors targeting class-IA PI3Ks, including PI3Ka and PI3K ß and their downstream pathways [17]. PIK3CA mutations can occur in any of the four domains of the gene: the adaptor-binding domain, C2 domain, helical domain and catalytic (kinase) domain. However, hot spot mutations occur mainly in exon 9 of the helical domain (E542 and E545) and in exon 20 of the kinase domain (H1047) [18]. AKT is an evolutionarily conserved kinase, also known as protein kinase B. Obviously, there are three members of the AKT family (AKT1-3), encoded by separate genes, but with over 80% amino-acid sequence identity which has both distinct and overlapping functions. AKT occupies a key regulatory node in the PI3K pathway, influencing significantly a wide range of cellular processes [19]. The AKT1 E17K mutation can activate the PI3K/AKT/mTOR pathway in a similar manner as PIK3CA alterations and promotes carcinogenesis through increasing cell proliferation or survival by phosphorylating several direct substrates that have crucial roles in cellcycle regulation. These substrates include cell cycle inhibitors and transcription factors [20, 21]. The present prospective study was planned to explore the mutational status of PIK3CA and AKT1 genes in BC biopsies and matched urines and to assess their use as anon-invasive molecular biomarker for patients´ diagnosis and monitoring.

## Methods

### Patients and study design

A total of 70 patients with bladder cancer were recruited at the Urology Department of the Military Hospital Mohammed V in Rabat - Morocco. Each biopsy was cut into two parts; one part was used for anatomy-pathological diagnosis and the other one was kept in -80°C for molecular biology explorations. Paired voided urine samples were obtained before surgery from all patients. Staging and grading were done according to the TNM classification (NMIBC includes Ta, T1, and Tis; and MIBC includes T2, T3, and T4) and World Health Organization criteria. Recurrence was defined as reappearance of primary NMIBC, with a lower or the same pathological stage, and progression was defined as a disease with a higher TNM stage upon relapse [9]. The study protocol was approved by the Ethics Committee for Biomedical Research, Faculty of Medicine and Pharmacy of Rabat - Morocco (Ref 82/19). All recruited patients have agreed to participate voluntarily in the study, been informed on the study and its objectives, and signed written consents. With respect to ethical rules, rights of confidentiality and anonymity of participants have been respected.

### Genomic DNA extraction

Genomic DNA was extracted from fresh frozen tumours and urine cell sediments using phenol/chloroform method [22]. All extracted DNA was quantified with a NanoDrop™ 2000 Spectrophotometer (Thermo Fisher Scientific, Waltham, MA, USA). DNA samples were used immediately or stored at -20°C until use.

### Evaluation of PIK3CA and AKT1 mutational status

Specific DNA primers were used to amplify exons 9 (F: AATCATCTGTGAATCCAGAGG; R: ATGCTGAGATCAGCCAAAAT) and 20 (F: CATTTGAGCAAAGACCTGAAGG; R: TGAGCTTTCATTTTCTCAGTTATC) of the PIK3CA gene and exon 1 of the AKT1 gene (F: TAGAGTGTGCGTGGCTCTCA; R: CTGAATCCCGAGAGGCCAA). DNA Amplification was performed on ProFlex PCR system (Applied Biosystems, Foster City, CA, USA) under the following conditions: 5 min at 95°C for initial denaturation, 35 cycles of 30s at 94°C for denaturation, 40s at specific annealing temperature of each primer set (54°C for PIK3CA exon 9, 58°C for PIK3CA exon 20 and 60°C for AKT1 exon 1 amplifications) and 40s at 72°C for DNA synthesis. At the end of the last cycle, a final elongation step of 7 min at 72°C was performed. PCR products were purified by ExS-Pure™ enzymatic PCR clean-up kit (NimaGen BV, Nijmegen, The Netherlands) and the sequencing was performed with BigDye®Terminator v3.1 Cycle Sequencing Kit (Applied Biosystems, Foster City, CA, USA), according to manufacturer´s protocol, on an ABI 3500 DNA analyzer (Applied Biosystems, Foster City, CA, USA). The obtained sequences were matched with the genes references sequences (Genes ID: 5290 and 207 for PIKCA and AKT1 respectively) collected from the GeneBank database. The sequence alignment was performed using Clustal W program in Bio-Edit Software.

### Statistical analysis

Statistical analyses were performed using IBM SPSS software version 23. The mutation status of studied genes in both tumours and urine samples was statistically evaluated using percentages and confidence intervals (CI) of 95%. The correlation between mutational status and clinico-histo-pathological parameters (tumours stage, grade, recurrence and progression) was evaluated using chi-squared test (χ^2^ test). Differences were considered significant at p values less than 0.05.

## Results

### Characteristics of the study population

Among the 70 patients with bladder cancer, 68 were male and 2 were female, with a sex ratio of 34. The mean age of patients was 67 years, ranging from 47 to 85 years. The clinico-pathological characteristics of tumour specimens are summarized in Table 1. Most cases were staged = PT1 (74.29%) and have high grade tumours (61.43%). In NMIBC, 23.08% of cases recurred and 9.62% progressed upon relapse (Table 1).

**Table 1:** tumour characteristics of people with bladder cancer

Parameter	Total cases	Percentage (%)
Gender		
Male	68	97.1
Female	2	2.9
Age		
<50	1	1.4
50-70	44	62.9
>70	25	35.7
Smoking		
Yes	28	40
No	42	60
Tumour stage		
≤PT1	52	74.3
>PT1	18	25.7
Tumour grade		
Low grade	27	38.6
High grade	43	61.4
Tumour recurrence		
Yes	12	23.1
No	40	76.9
Tumour progression		
Yes	5	9.6
No	47	90.4

### Evaluation of the mutational status of PIKCA and AKT1 genes in bladder biopsies

AKT1 alterations were poorly detected in our cohort and were obtained in only in 1.4% of samples (1/70).AKT1 E17K mutation was found in a man with pT1 stage and high-grade tumour (Table 2). Mutations in PIK3CA gene were found in 12.6% of patients (9/70). In PIK3CA exon 9, 2 point mutations, E545K and Q546E, were reported in 3 and 1 cases, respectively, and a SNP (E547E) was reported in 1 case. In PIK3CA exon 20, 2 point mutations, L989V and H1047R, were found in 1 and 2 cases, respectively. Moreover, I1022I SNP was found in 2 cases and T1025T SNP in 1 case. Of note, 2 cases have 2 point mutations each; the first one have 1 SNP and 1 point mutation (E547E/Q546E) in exon 9 and the second harboured 2 SNPs (I1022I/T1025T) in the exon 20. The relationship between mutations and patient clinico-phatological features is summarized in Table 2, and clearly showed that mutations of PIK3CA were mainly observed in low tumour stages (= pT1), reported in 15.4 % of cases (8/52), and in high grade tumours, reported in 14% of cases (6/43). Of particular interest, PIK3CA and AKT1 point mutations weren´t detected in any recurrent or progressive case (Table 2). Statistical analysis showed no significant association between the studied AKT1 and PIK3CA point mutations and patients´ clinico-pathological parameters (p > 0.05) (Table 2).

**Table 2:** distribution of PIK3CA and AKT1 genes mutations according to clinicopathological parameters

Parameter	PIK3CA		AKT1	
	Overall n=9		Overall n=1	
	+	-	+	-
**Gender**				
Male	9	59	1	67
Female	0	2	0	2
P value	0.999		1	
**Age**				
< 50	0	1	0	1
50 -70	7	36	1	43
> 70	2	23	0	25
P value	0.703		0.410	
**Smoking**				
Yes	3	25	1	27
No	6	36	0	42
P value	0.525		0.997	
**Tumour stage**				
≤ PT1	8	44	1	51
>PT1	1	17	0	18
P value	0.998		0.998	
**Grade**				
LG	3	24	0	27
HG	6	37	1	42
P value	0.512		0.998	
**Recurrence**				
Yes	0	12	0	12
No	9	31	1	39
P value	0.999		0.999	
**Progression**				
Yes	0	5	0	5
No	9	38	1	46
P value	0.999		0.999	

### Evaluation of the mutational status of PIK3CA and AKT1 genes in matched urine sediments

In urine sediments, PIK3CA and AKT1 gene mutations were reported in 8.6% (6/70) and 1.4% (1/70) of samples, respectively. Comparison between mutational status of the studied genes in bladder biopsies and paired urine sediments DNA was assessed and is reported in Table 3. Interestingly, all cases showing mutations in urine sediments have been diagnosed with BC after haematuria episode and have aberrant mutations in the corresponding DNA from bladder biopsies, and reciprocally, no mutation was detected in urine sediments of patients showing the absence of point mutations in bladder biopsies, suggesting a specificity of mutation detection in urine sediments of 100% for both AKT and PIK3CA genes. Point mutation detection in urine sediments showed high sensitivity for AKT1 gene (100%); the only point mutation obtained in AKT1 gene was reported in both patient´s biopsy and urine samples. Moderate mutation detection sensitivity in urine samples was obtained for PIK3CA gene (66.7%); point mutations were detected in only 6 urine sediments samples among the 9 patients who harboured point mutations in the corresponding biopsies. Additionally, all mutated cases have been diagnoses with bladder cancer after presenting hematuria episode (Table 3).

**Table 3:** comparison between mutational status in bladder biopsies and voided urine sediments

Genes	Mutations in fresh biopsies		Mutations in urine sediments		Sensitivity % (95% CI)	Specificity % (95% CI)	Hematuria % (95% CI)
	N	% (95% CI)	N	% (95% CI)			
PIK3CA	9	12.6 (6.1-23)	6	8.6 (3.2-17.7)	66.7 (29.9-92.5)	100 (94.9-100)	100 (66.4-100)
AKT1	1	1.4 (0-7.7)	1	1.4 (0-7.7)	100 (2.5-100)	100 (94.9-100)	100 (2.5-100)

CI = confidence interval. The sensitivity reflects the fraction of patients in which the urine DNA was mutated among cases with mutation in matched tumour DNA of the same genes.

## Discussion

During the last decades, a great interest was given to the molecular events in bladder tumorigenesis as determinant of cancer progression, and worldwide published data have identified molecular events that could be of interest in patients´ diagnosis, prognosis and target therapy [23, 24]. In the present study, mutation of AKT1 gene is a rare event in bladder cancer in Moroccan patients. Among 70 patients, only 1.4% (1/70) harboured the E17K point mutation. This results is in agreement with reported finding in breast cancer (4.3%) [25], ovarian 2% and colorectal 6% cancers [26]. Therapies targeting AKT1 mutated cancers showed that treatment with AZD5363, an ATP-competitive pan-AKT kinase inhibitor, yielded durable responses and tumour regressions across a variety of tumour types harbouring the E17K mutation including breast, endometrial, cervical, and lung cancers. Mutations as D323H in AKT1 and W80C in AKT2 are relatively resistant to the allosteric AKT inhibitor MK-2206 suggesting that the vast majority of rare AKT variants are passenger mutations with no effect on drug sensitivity [27, 28].

PIK3CA mutations of the helical domain has been reported in up to 26% of breast cancers, E542K and E545K being the most prevalent point mutations and were reported in 20% and 11% of cases, respectively [29]. In our study, PIK3CA mutations were found in 12.6% (9/70) cases distributed in exon 9 (3 E545K, 1 Q546E and 1 E547E) and 20 (1 L989V; 2 I1022I;1 T1025T and 2H1047R). Interestingly, among the 9 positive cases, 2 harboured 2 point mutations; 1 case has Q546E point mutation and E547E SNP in exon 9 and the second carried the I1022I and T1025T SNPs. In a cohort study of 61 patients, with two proliferative skin lesions lacking malignant potential, the typical cancer-associated PIK3CA mutations E542K, E545K and H1047R were revealed in 16% of the cases [30]. In this study, the H1047R mutation affecting the catalytical domain of PIK3CA gene was found only in two patients supporting the widely reported data showing that PIK3CA alterations affecting the catalytical domain mutations, includingH1047R, were relatively rare (16.7%), whereas mutations affecting the helical domain, including E545K/D and E542K, prevail and are reported in relatively high number of cases [31]. In urothelial tumours somatic mutations in PIK3CA gene may be of use for early detection of primary and recurrent tumours in urine-based assays, not only for prognosis prediction, but also as molecular biomarkers for targeted therapies [32]. Specific knockdown of PIK3CA inhibited proliferation, migration, anchorage-independent growth and in vivo tumour growth of cells with PIK3CA mutations [15]. Of particular interest, E542K and E545K were respectively identified in two cell lines: BLCAb001 and BLCAb002. BLCAb001 is less responsive to cisplatin than BLCAb002, suggesting that the presence of activating PIK3CA mutations may not necessarily predict in vivo treatment response to PI3K targeted therapies, while specific gene alterations may be predictive for cisplatin response in BC models and, potentially, in patients as well [33].

In BC, numerous studies have highlighted the possibility to detect bladder oncogene mutations in DNA isolated from urine sediments, giving the opportunity to use a non-invasive approach for genetic studies and molecular diagnosis [34]. Of particular interest, it was shown that assessment of some genetic alterations in urine sediments adds diagnostic value to urine cytology and confers high sensitivity and specificity for the detection of urothelial carcinoma [35]. In this context, we have evaluated the mutational status of PIK3CA and AKT1genes in bladder cancer biopsies and matched urines, and assess their use as anon-invasive molecular biomarker for patients´ diagnosis and monitoring. The use of DNA obtained from urine sediment; as template to detect mutations, provides sensitivities of 66.7% and 100% for PIK3CA and AKT1 genes, respectively. No false-positive result was detected and the mutations in exfoliated cells were observed only in cases showing mutations in corresponding tumours, giving a specificity of 100% for both PIK3CA and AKT1 genes. Sensitivity of detecting mutations of PIK3CA gene in urine sediments is equal to 66.7% and the only 1 mutation in AKT was reported in both the patient´s biopsy and voided urine sediment. These results must be considered with a lot of precaution, as the number of positive cases is low. In Morocco, as in most developing countries, the treatment of BC is based on the clinico-pathologic features, and the main challenge faced by clinicians is to overcome the treatment inefficiency and prevent the development of disease´ recurrence and/or resistance to chemo and/or radiotherapy [36, 37]. The main limitation of the study is the low number of cancer cases that will not reflect the real mutational status of these genes in BC and consequently will not be useful for better assessment of the association between the mutational status and clinico-pathological features.

## Conclusion

In conclusion, this study is very informative and clearly showed that mutations in AKT1 and PIK3CAare rare events and could not be considered as valuable biomarkers for bladder cancer management. Other studies are needed to explore the impact of these mutations in the overall PI3K-AKT-mTOR signalling pathway and bladder cancerogenesis.

### What is known about this topic

- *PI3K-AKT-mTOR signalling pathways play a major role in tumour growth, proliferation and cellular survival;*
- *Urine cytology has a low detection sensitivity, especially for low-grade tumours*.

### What this study adds

- *AKT1 mutation is a rare event in bladder cancer;*
- *Detecting mutations in urine sediment is a promising and reliable method for non-invasive diagnosis and monitoring*.
